# Expression of *μ*-protocadherin is negatively regulated by the activation of the *β*-catenin signaling pathway in normal and cancer colorectal enterocytes

**DOI:** 10.1038/cddis.2016.163

**Published:** 2016-06-16

**Authors:** L Montorsi, S Parenti, L Losi, F Ferrarini, C Gemelli, A Rossi, G Manco, S Ferrari, B Calabretta, E Tagliafico, T Zanocco-Marani, A Grande

**Affiliations:** 1 Department of Life Sciences, University of Modena and Reggio Emilia, Modena, Italy; 2 SOFAR S.p.A., Milano, Italy; 3 Department of Surgical, Medical, Dental and Morphological Sciences with Interest in Transplant, Oncological and Regenerative Medicine, University of Modena and Reggio Emilia, Modena, Italy; 4 Department of Clinical and Diagnostic Medicine and Public Health, University of Modena and Reggio Emilia, Modena, Italy; 5 Department of Cancer Biology and SKKC, Thomas Jefferson University, Philadelphia, PA, USA; 6 Department of Medical and Surgical Sciences, University of Modena and Reggio Emilia, Modena, Italy; 7 Center for Genome Research, University of Modena and Reggio Emilia, Modena, Italy

## Abstract

Mu-protocadherin (MUCDHL) is an adhesion molecule predominantly expressed by colorectal epithelial cells which is markedly downregulated upon malignant transformation. Notably, treatment of colorectal cancer (CRC) cells with mesalazine lead to increased expression of MUCDHL, and is associated with sequestration of *β*-catenin on the plasma membrane and inhibition of its transcriptional activity. To better characterize the causal relationship between *β*-catenin and MUCDHL expression, we performed various experiments in which CRC cell lines and normal colonic organoids were subjected to culture conditions inhibiting (FH535 treatment, transcription factor 7-like 2 siRNA inactivation, Wnt withdrawal) or stimulating (LiCl treatment) *β*-catenin activity. We show here that expression of MUCDHL is negatively regulated by functional activation of the *β*-catenin signaling pathway. This finding was observed in cell culture systems representing conditions of physiological stimulation and upon constitutive activation of β-catenin in CRC. The ability of MUCDHL to sequester and inhibit *β*-catenin appears to provide a positive feedback enforcing the effect of *β*-catenin inhibitors rather than serving as the primary mechanism responsible for *β*-catenin inhibition. Moreover, MUCDHL might have a role as biomarker in the development of CRC chemoprevention drugs endowed with *β*-catenin inhibitory activity.

Cadherins are integral membrane proteins constituting a complex superfamily, which consists of >100 members and are divided into classical cadherins and protocadherins. The latter are characterized by the presence of one or more unconventional domains in addition to the typical cadherin-like domains, which are responsible for the canonical function of these proteins.^1^ From a structural point of view, cadherins are composed of an extracellular portion, harboring its cadherin-like domains through which they promote intercellular adhesion, and an intra-cellular portion, through which they inhibit cell proliferation. This effect is achieved by sequestering *β*-catenin in a submembrane location, thereby preventing its translocation to the nucleus where, in normal conditions, it activates the transcription of growth-promoting target genes.^2^ Not surprisingly, as a result of these activities, cadherins exert a recognized onco-suppressor effect that can be observed in different cell types, depending on their expression pattern.^3^ In the last decade, a novel cadherin, named *μ*-protocadherin (MUCDHL) to underline the hybrid nature of its extracellular portion, which contains four cadherin-like and three mucin-like domains, has been isolated and characterized.^4^ This protein maintains all the general features of cadherins and carries, in its intra-cellular portion, four prolin-rich domains together with a single PDZ domain, previously shown to bind *β*-catenin. A survey of MUCDHL distribution revealed that significant levels of its expression were exclusively detected in a couple of anatomical sites, including colorectal mucosa.^5^ This observation suggested a possible role in colorectal carcinogenesis that was subsequently supported by experimental results. Indeed, interaction of MUCDHL with *β*-catenin, which is constitutively activated in colorectal cancer (CRC), was confirmed and MUCDHL expression exhibited a remarkable downregulation in >70% of CRC cases, in association with higher proliferation indexes.^6^ Notably, data obtained in our laboratory showed that treatment of CRC cells with an anti-inflammatory drug named mesalazine (5-ASA), that is able to inhibit *β*-catenin signaling *in vitro* and to exert anti-CRC chemoprevention activity *in vivo*,^7^ markedly induces MUCDHL expression.^8^ This finding might, in our opinion, reflect two distinct explanations: (i) 5-ASA could induce MUCDHL expression, sequester *β*-catenin on the cell membrane and inhibit its signaling pathway; or (ii) 5-ASA could inhibit *β*-catenin, leading to an upregulation of MUCDHL, dependent on the fact that expression of this protein is negatively regulated by *β*-catenin signaling (Supplementary Figure 1). In other terms, upregulation of MUCDHL may represent the cause or the effect of *β*-catenin inhibition, respectively. In both cases MUCDHL would, once expressed, sequester and inhibit *β*-catenin on the plasma membrane, although with different biological consequences. To better understand the correlation between *β*-catenin activity and MUCDHL expression, we performed experiments in which the *β*-catenin signaling pathway was inhibited in CRC cell lines using a chemical inhibitor or a siRNA-based approach and showed that MUCDHL expression is upregulated by both treatments. By contrast, the opposite effect was observed in human colonic organoids treated with a compound able to activate the *β*-catenin signaling pathway.

## Results

### Downregulation of MUCDHL expression correlates with APC mutation in sporadic CRC

**Figure 1:**
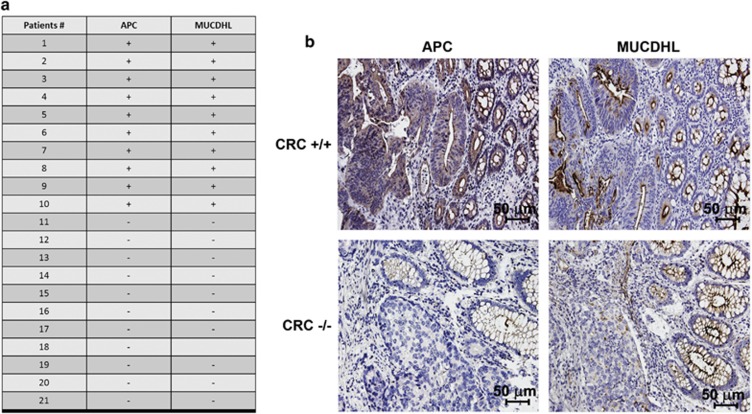
(**a**) Table summarizing the results of the immuno-histochemical analysis of APC and *μ*-protocadherin (MUCDHL) protein levels in 21 CRC samples; + and − indicate presence or absence of the analyzed protein, respectively. (**b**) Immuno-histochemical analysis of two representative CRC cases exhibiting a double positive (APC+/MUCDHL+) (CRC +/+) or a double negative (APC−/MUCDHL−) (CRC −/−) expression pattern. Positivity appears as a dark staining; cell nuclei were counterstained with hematoxylin

An important clue supporting the possibility that regulation of MUCDHL expression is a downstream event of the *β*-catenin signaling pathway (second hypothesis of Introduction) came from the observation that MUCDHL downregulation always accompanies adenomatous polyposis coli (APC) inactivation in CRC. In fact, immuno-histochemical data obtained in our laboratory showed that 11 out of 21 analyzed CRC cases were negative for MUCDHL and APC expression, whereas the remaining exhibited a double positivity for the same proteins (Figure 1). This finding suggests that the constitutive activation of the *β*-catenin signaling pathway, determined by mutation/inactivation of APC, is responsible for a reduced expression of MUCDHL, implying that the latter is negatively regulated by the former.

### Treatment of CaCo2 and HCT116 CRC cell lines with a *β*-catenin inhibitor leads to increased expression of MUCDHL

**Figure 2:**
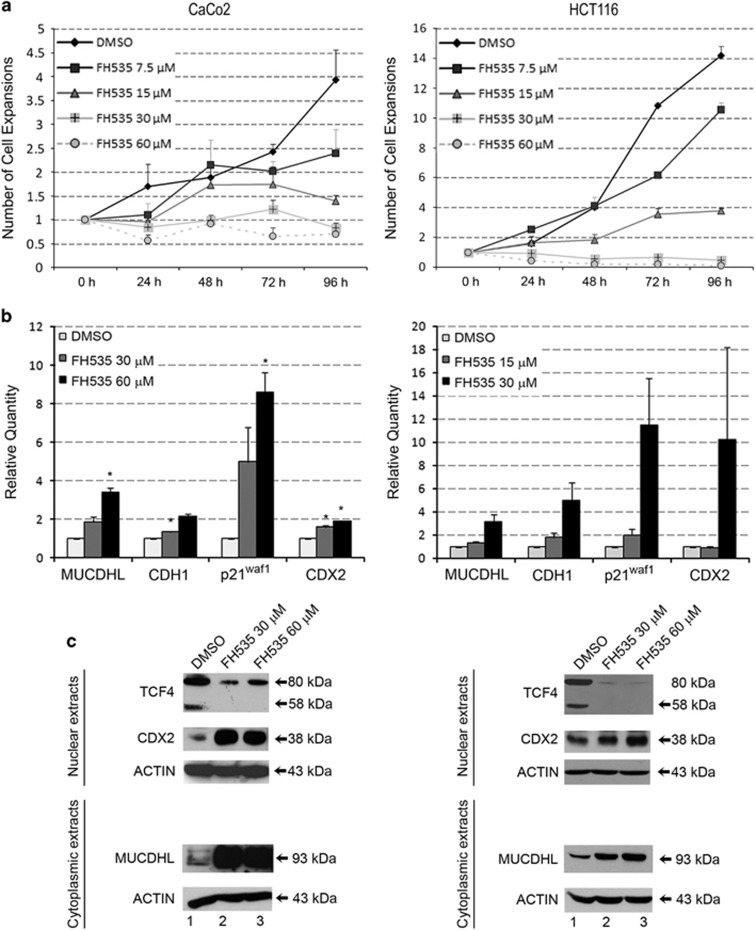
Effects of FH535 treatment in the CaCo2 and HCT116 CRC cell lines. (**a**) FH5353 causes a dose- and time-dependent inhibition of cell proliferation. Cells were treated with the indicated concentrations of FH535 and counted after 24, 48, 72 and 96 h (*x* axis). Number of cell expansions are reported on *y* axis as mean values obtained from three independent experiments. (**b**) FH535 induces upregulation of *μ*-protocadherin (MUCDHL), E-cadherin (CDH1), p21^waf1^ and CDX2 transcripts. Cells were treated with the indicated doses of FH5353 and analyzed by QRT-PCR after 96 h for CaCo2 and 48 h for HCT116 cells. Mean variations of mRNA expression levels, deriving from three independent experiments, are reported in the *y* axis as relative fold-change (relative quantity). Results are represented as mean±S.E.M. values and asterisks indicate statistically significant results (*P*<0.05). (**c**) Western blot analysis following treatment with FH535 shows a decreased expression of TCF4 protein and a concomitant increase of MUCDHL and CDX2 protein expression. Cells were treated with the indicated doses of FH535 and analyzed after 96 h for CaCo2 and 48 h for HCT116 cells. This analysis was performed on nuclear and cytoplasmic extracts and normalized with actin. Analyzed proteins and their molecular weights are indicated on the left and on the right, respectively

To further support the hypothesis that expression of MUCDHL is negatively regulated by active *β*-catenin, we treated the CaCo2 and HCT116 CRC cell lines with the FH535 compound, which inhibits the *β*-catenin pathway through complete silencing of the *β*-catenin-regulated transcription factor 7-like 2 (TCF4) transcription factor. Cell counts, obtained in a preliminary dose-response experiment, indicated that a complete inhibition of proliferation was reached with a 30–60 *μ*M concentration after 96 h of treatment, in CaCo2 cells, and 15–30 *μ*M concentration after 48 h of treatment, in HCT116 cells (Figure 2, panel a; Supplementary Table 1 and 2). Thus, under these experimental conditions, we evaluated changes in gene expression at the mRNA and protein level, by quantitative real-time PCR (QRT-PCR) and western blot, respectively, of MUCDHL, E-cadherin (CDH1), previously characterized as a negative target of *β*-catenin, p21^waf-1^, as a molecular marker of proliferation arrest, and caudal type homeobox transcription factor 2 (CDX2), a transcriptional activator of MUCDHL.

In the CaCo2 cell line, at the highest FH535 concentration, transcript levels of the four analyzed genes were induced 3.4-, 2.2-, 8.6- and 1.9-fold, respectively, with virtually all variations exhibiting statistical significance (Figure 2, panel b; Supplementary Table 3). A similar response was observed in the HCT116 cell line where the induction was 3.2, 5.0, 11.5 and 10.3 fold, although the changes did not reach statistical significance (Figure 2, panel b; Supplementary Table 4).

As expected, protein levels of TCF4 were strongly downregulated in both FH535-treated CRC cell lines and, in general, gene expression changes detected by western blot were more pronounced than those observed by QRT-PCR (Figure 2, panel c). Indeed, at the highest FH535 concentration, the upregulation of MUCDHL and CDX2 proteins was 11.3 and 6.9 times in CaCo2 cells and 3.0 and 2.5 times in HCT116 cells.

Together, this analysis suggests that the *β*-catenin signaling pathway exerts a negative regulation on the expression of MUCDHL and CDX2.

### Transfection of CaCo2 and HCT116 CRC cell lines with an anti-TCF4-specific siRNA leads to increased expression of MUCDHL

To confirm the data obtained with the FH535 compound using a more specific approach, the same cell lines were transfected with a mix of three TCF4 siRNA oligonucleotides and the effects of this treatment on gene expression were evaluated by QRT-PCR and western blotting.

**Figure 3:**
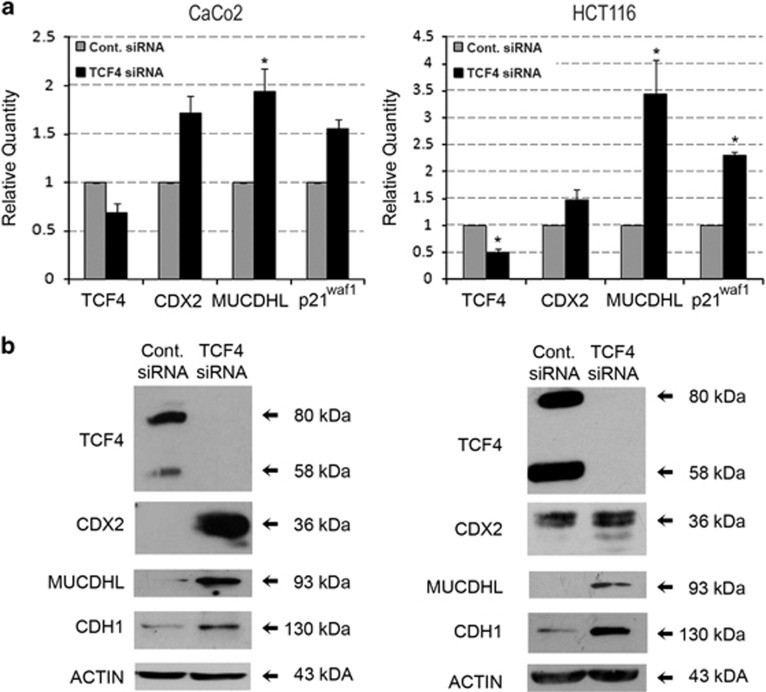
Inhibition of *β*-catenin pathway induced by transfection of an anti-TCF4-specific siRNA in CaCo2 and HCT116 cell lines. (**a**) siRNA-mediated silencing of TCF4 results in the induction of MUCDHL, CDX2, and p21^waf1^ transcripts. Cells were transfected with a scrambled siRNA (Cont. siRNA) or with a mix of siRNA against TCF4 (TCF4 siRNA) and analyzed after 72 h by QRT-PCR. Data are presented as in Figure 2, panel **b**. Results are represented as mean±S.E.M. values and asterisks indicate statistically significant results (*P*<0.05). (**b**) Western blot analysis confirming the results of the QRT-PCR reactions. This analysis was performed on cell samples under the same experimental conditions and the results presented as explained in Figure 2, panel **b**

Transfection with the anti-TCF4 siRNA induced an apparently modest downregulation of its target TCF4 mRNA of 32 and 50% in CaCo2 and HCT116 cells, respectively, as assessed by QRT-PCR (Figure 3, panel a; Supplementary Table 5 and 6); however, western blot analysis indicated a complete disappearance of the corresponding protein in both cell lines (Figure 3, panel b), probably reflecting the combined effect of increased RNA degradation and translation inhibition induced by the anti-TCF4 siRNA on its target.

QRT-PCR analysis revealed that all other tested genes were upregulated upon TCF4 silencing, almost always with statistically significant *P-*values (Figure 3, panel a; Supplementary Table 5 and 6). Among them, MUCDHL exhibited the most pronounced variation, showing a 1.9-fold increase in CaCo2 cells and 3.4 increase in HCT116 cells. The results of western blot analysis confirmed those of QRT-PCR (Figure 3, panel b).

### Withdrawal of Wnt stimulation in human colonic organoids induces MUCDHL expression

**Figure 4:**
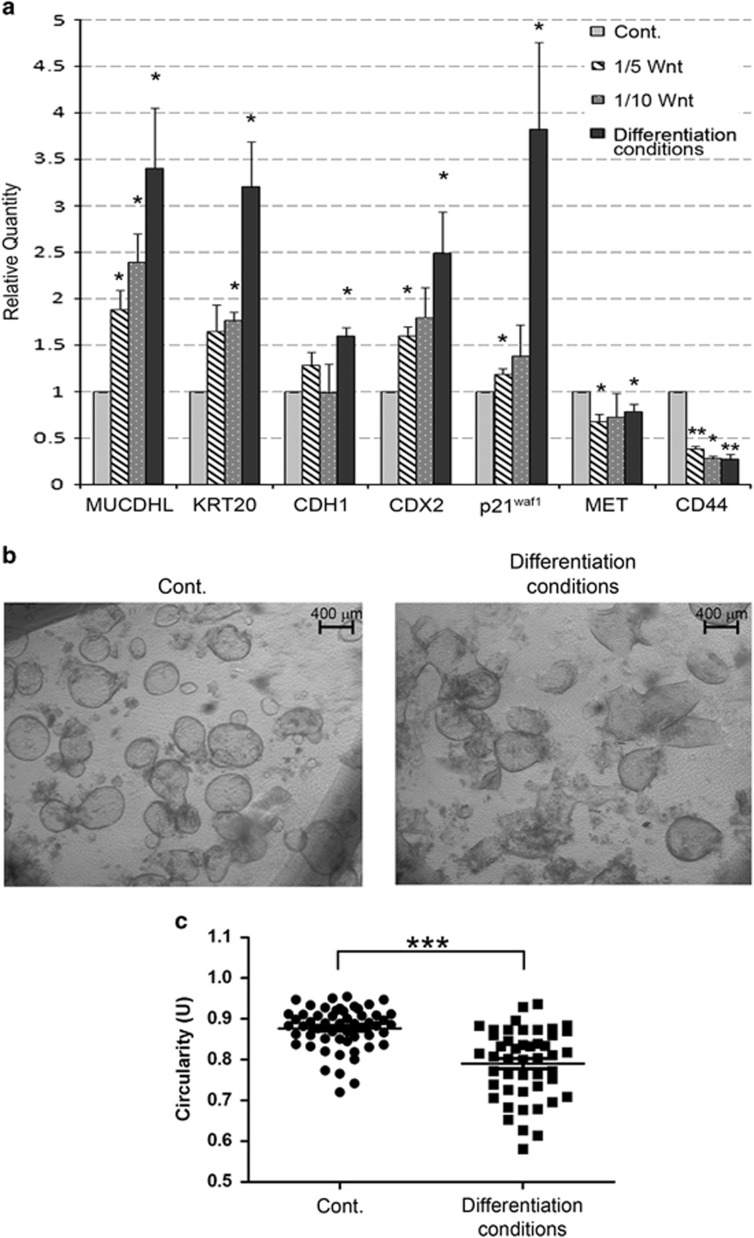
Deprivation of WNT3a in culture medium of human colonic organoids induce the mRNA expression of MUCDHL and CDX2. (**a**) Colonic organoids were cultured in the presence of gradually reduced concentrations of WNT3a (1/5 and 1/10 of the standard concentration) or under differentiation-inducing conditions characterized by the complete withdrawal of all *β*-catenin agonists from culture medium. QRT-PCR analysis was performed after 72 h and the mean values obtained by four independent experiments presented as in previous figures. Results are represented as mean±S.E.M. values and asterisks indicate statistically significant results (*P*<0.05). (**b**) Representative bright-field image of colonic organoids grown under standard (Cont.) or differentiation conditions. (**c**) Analysis of circularity performed on colonic organoids cultured under standard (Cont.) or differentiation conditions. The dot plot shows the results of two independent experiments represented as mean±S.E.M. (***P<0.0001)

To further confirm the data obtained in CaCo2 and HCT116 cell lines in a primary cell context, we generated in our laboratory the experimental model of human colonic organoids previously described by Clevers and collaborators.^9^ Inhibition of the *β*-catenin pathway was then mimicked through scalar reduction of the Wnt ligand that was added to the culture at a standard dose, and at one-fifth and one-tenth concentrations. A control sample in which organoids were induced to differentiate was also set up using, besides the 90% Wnt reduction, complete withdrawal of other *β*-catenin agonists such as PGE2. In addition to the genes tested above, QRT-PCR analysis included the Keratin-20 gene, as a marker of enterocyte differentiation, and the Met and CD44 genes, as direct targets of the *β*-catenin signaling pathway. As shown in Figure 4a and Supplementary Table 7, mRNA expression of MUCDHL, Keratin-20, E-cadherin, CDX2 and p21^waf1^ genes, showed a gradual increase from standard culture to differentiation conditions, reaching mean values of 3.4, 3.2, 1.6, 2.5, 3.8, respectively. The Met and CD44 genes showed an exactly inverted trend, exhibiting a 21 and 73% decrease, respectively, of their mRNA expression. Most comparisons were statistically significant (Figure 4, panel a; Supplementary Table 7). Inhibition of the β-catenin signaling also affected the morphology of organoids, revealing a collapsed shape evidenced by light-inverted microscope examination followed by the assessment of circularity values with the ImageJ software (0.88±0.007 U of control versus 0.79±0.012 U of differentiated samples, *P*<0.0001) (Figure 4, panels b and c, Supplementary Table 10).

### Treatment of human colonic organoids with LiCl inhibits MUCDHL expression

**Figure 5:**
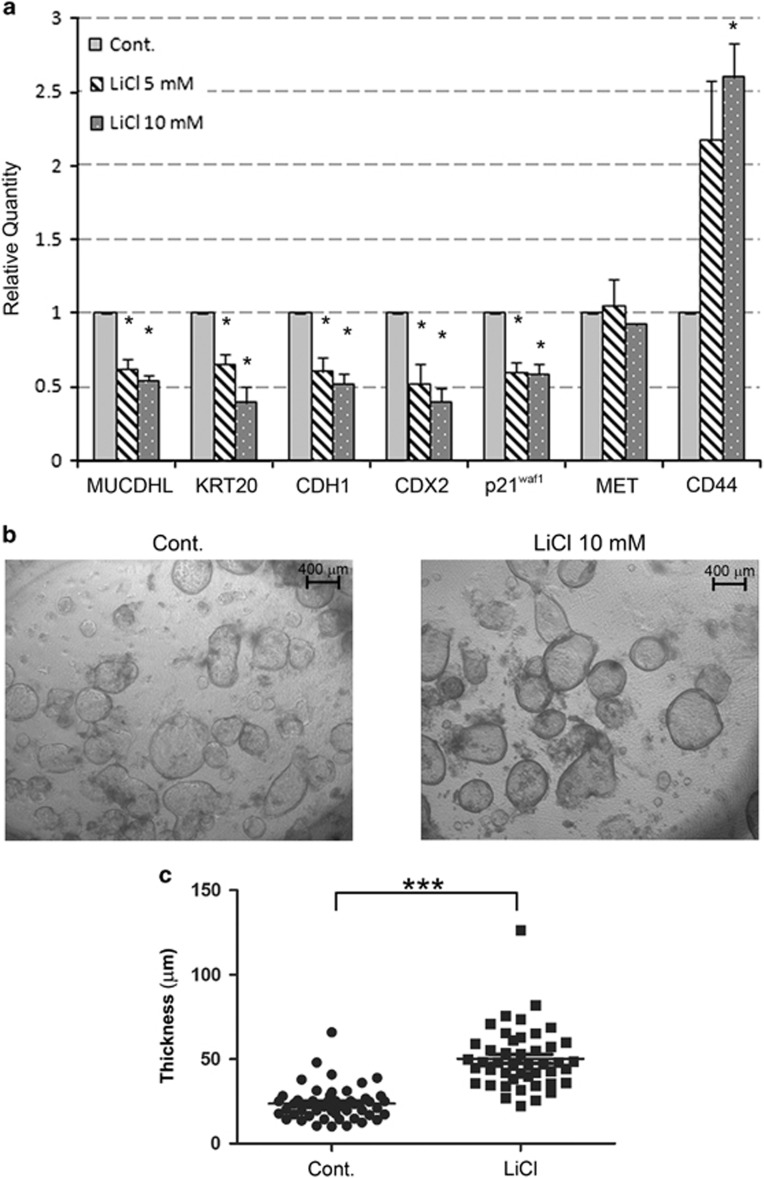
Stimulation of the *β*-catenin signaling pathway in human colonic organoids, with a GSK3*β* inhibitor, reduces MUCDHL and CDX2 mRNA expression. (**a**) Colonic organoids were treated with the indicated amount of LiCl and analyzed by QRT-PCR 72 h later. Mean values obtained from four independent experiments are shown as in previous figures. Results are represented as mean±S.E.M. values and asterisks indicate statistical significance (*P*<0.05). (**b**) Representative bright-field image of colonic organoids grown under standard conditions (Cont.) or in presence of LiCl 10 mM. (**c**) Analysis of epithelial lining thickness of colonic organoids grown under standard conditions (Cont.) or in presence of LiCl. The dot plot shows the results of two independent experiments represented as mean±S.E.M. (***P<0.0001)

The experimental advantage of human colonic organoids is also represented by the possibility to analyze the effects of inducible stimulation of *β*-catenin signaling, unlike the CRC cell lines where this pathway is constitutively activated as consequence of APC mutation. This condition was obtained upon treatment with 5 or 10 mM LiCl, which is known to inhibit the GSK3*β* enzyme, leading to stabilization and activation of *β*-catenin. Then, QRT-PCR analysis was carried out as described in the previous experiment. With the only exception of the Met gene, the results observed were, as expected, opposite to those elicited by withdrawal of the *β*-catenin agonists. In fact, at the highest LiCl concentration, there was a 46% decrease for MUCDHL, 61% for Keratin-20, 48% for E-cadherin, 60% for CDX2 and 42% for p21^waf1^, whereas, under the same conditions, the CD44 gene showed a 2.6-fold increase of its transcript levels (Figure 5, panel a; Supplementary Table 8). All variations were statistically significant. In agreement with its activity on the β-catenin pathway, treatment with LiCl determined a positive effect on the proliferation rate of organoids demonstrated by a more pronounced thickness of their peripheral lining, observed again through a light-inverted microscope and measured with the ImageJ software (50.2±2.7 *μ*m of LiCl treated versus 23.7±1.5 *μ*m of control samples, *P*<0.0001) (Figure 5, panels b and c; Supplementary Table 11).

### CDX2 silencing abolishes MUCDHL upregulation induced by 5-ASA treatment

In the studies presented here, variations of MUCDHL expression in response to modulators of *β*-catenin activity, correlate with those of the CDX2 transcription factor, suggesting its involvement in the regulation of MUCDHL levels. To verify this possibility, the CaCo2 cells were transfected with a scrambled control siRNA or with a mix of three anti-CDX2 siRNAs for 12 h, and then cultured for an additional 4-day period in the absence or presence of 20 mM 5-ASA. The decision to use this compound was based on its anti-CRC chemoprevention capacity, previously related to its ability to inhibit *β*-catenin activity and induce MUCDHL expression.

**Figure 6:**
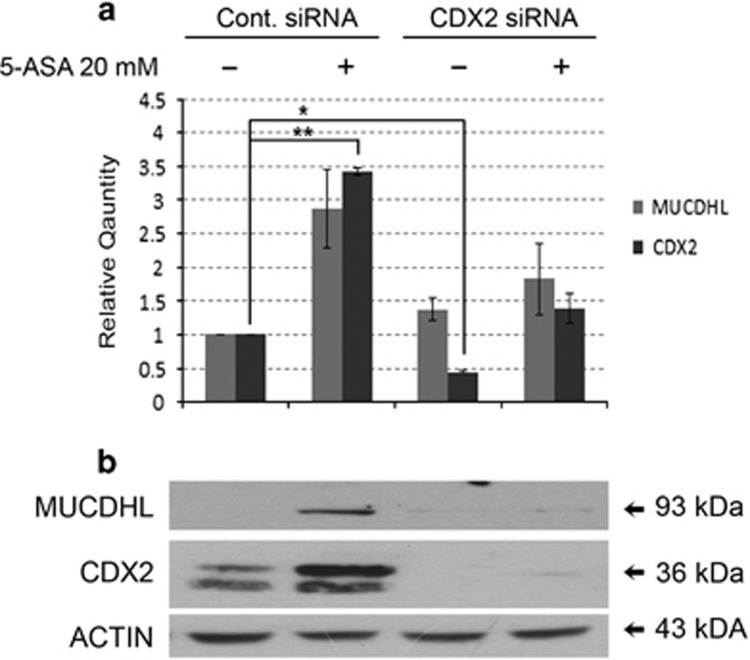
Transfection of CaCo2 cells with an anti-CDX2 siRNA interferes with upregulation of *μ*-protocadherin expression induced by stimulation with 5-ASA. (**a**) CaCo2 cells were transfected with a scrambled siRNA (Cont. siRNA) or a mix of anti-CDX2 siRNAs (CDX2 siRNA) and stimulated with 5-ASA to induce *μ*-protocadherin expression. The effect determined on the expression of CDX2 and MUCDHL mRNAs was then analyzed by QRT-PCR. Results are represented as mean±S.E.M. values and asterisks indicate statistically significant results (*P*<0.05). (**b**) The effect determined on the expression of the same genes at the protein level was evaluated by western blot analysis. Data were presented according to the same modalities of the previous figures

As expected, CaCo2 cells transfected with control siRNA and exposed to 5-ASA, exhibited a 2.8-and 3.4-fold increase of CDX2 and MUCDHL mRNA, respectively, detected by QRT-PCR (Figure 6, panel a, Supplementary Table 9). Instead, transfection of the anti-CDX2 siRNAs resulted in ~50% reduction of these values returning them approximately to their basal level. Western blot analysis, carried out under the same experimental conditions, showed that CDX2 silencing was complete and variations of MUCDHL protein were consistent with those of its transcript (Figure 6, panel b).

## Discussion

Metastasis is the result of a complex process by which cancer cells detach from the initial tumor mass and acquire the capacity to migrate to distinct anatomical sites where they give rise to one or more secondary tumors. Reduced expression of cadherins is critically important in this process, owing to their role in promoting cell–cell adhesion in normal conditions.^10^ E-cadherin is considered the main adhesion molecule in epithelial tissues and loss of its expression, when observed, has been ascribed to epigenetic alterations (gene promoter hypermethylation) or to transcriptional repression mediated by Snail family members.^11^ These proteins can be divided into two subgroups that are able to repress E-cadherin expression directly, binding to its promoter region (Snail, Slug), or indirectly, with a different mechanism (Twist, ITF2). Interestingly, a number of reports have highlighted the ability of the *β*-catenin pathway to activate Snail family transcription repressors.^12, 13^ This effect is reached through a couple of distinct molecular mechanisms: (i) activation of Snail and Slug as consequence of Wnt-induced GSK3*β* inhibition, leading to their stabilization and nuclear translocation; and (ii) direct transcriptional activation of Twist and ITF2 by *β*-catenin/TCF4.^14, 15, 16, 17^ However, experimental evidence supporting the existence of a molecular cascade, linking *β*-catenin signaling to Snail proteins and to E-cadherin expression, has almost exclusively been obtained in breast cancer and its normal counterpart.^14, 15^ On the other hand, loss of E-cadherin expression is rarely detected in CRC where downregulation of MUCDHL is, instead, a common event.^6^ In this report, we clearly demonstrated, for the first time, that MUCDHL expression is negatively regulated by *β*-catenin signaling. This finding was obtained in epithelial cancer cells as well as in normal colon enterocytes, represented by CRC cell lines and colonic organoids, respectively. Moreover, treatments inhibiting the entire *β*-catenin pathway (Wnt deprivation), or GSK3*β* activity (LiCl), or *β*-catenin-dependent transcription (FH535, siRNA), all affected MUCDHL expression, suggesting that this effect could be ascribed to a transcription repressor induced by *β*-catenin, such as the ITF2 member of the Snail family. Computational analysis of ‘Chip-Seq' data contained inside the ENCODE project database, allowed us to retrieve, among Snail family proteins able to bind MUCDHL promoter, the Zeb1 repressor. However, QRT-PCR analysis on HCT116 cells transfected with an anti-TCF4 siRNA oligonucleotide or treated with the FH535 compound, failed to detect an upregulated expression of genes coding for either ITF2 or Zeb1, suggesting that they do not have an important role in *β*-catenin regulation of MUCDHL expression (data not shown). Conversely, variations of MUCDHL expression, in response to stimuli modulating the activity of the *β*-catenin pathway, consistently showed a direct correlation with those of the transcription activator CDX2. A previous report has demonstrated that the transcription of MUCDHL gene is directly controlled by this transcription factor through a specific DNA-binding site in its promoter region.^18^ Taken together, these observations allow us to speculate that the negative regulation of MUCDHL expression by *β*-catenin might be actually exerted via CDX2, through a transcription repressor that remains to be identified. This hypothesis is supported by the evidence that CDX2 silencing interferes with the capacity of 5-ASA, a *β*-catenin inhibitor with anti-CRC chemoprevention activity, to upregulate MUCDHL expression. In aggregate, our data suggest that the silencing of MUCDHL expression observed in CRC is consequent to the constitutive activation of the *β*-catenin pathway, which is typical of these tumors. The development of compounds able to restore the expression of MUCDHL, thus stimulating its anti-tumor (anti-proliferative and anti-metastatic) properties, opens important possibilities for chemoprevention and/or therapy of CRC. The rationale underlying this pharmacological approach also implies a possible role of MUCDHL as biomarker to estimate the extent to which these compounds may be effective. The negative effect on MUCDHL expression exerted by constitutive *β*-catenin activation observed in CRC may be simply the consequence of a physiological mechanism already operating in normal colon enterocytes. Based on the capacity of MUCDHL to sequester and inhibit *β*-catenin at the cell membrane, this biological activity might represent the result of a positive feedback amplifying the final effects of stimuli that either activate or inhibit the *β*-catenin signaling pathway. The inverse correlation existing along the axis of colon crypt between the nuclear localization of *β*-catenin (observed at the bottom) and the expression of MUCDHL (detected on the top), in our opinion, provides further support for this hypothesis.

## Materials and Methods

### Cell cultures and treatments

HCT116 and CaCo2 cells were obtained from ATCC (Rockville, MD, USA) and cultured in DMEM supplemented with 10% fetal bovine serum (Sigma-Aldrich, St. Louis, MO, USA), 2 mM-Glutamine (EuroClone, Devon, UK) and 100 U/ml Penicillin/Streptomycin (EuroClone).

FH535 was purchased from Sigma-Aldrich and dissolved in DMSO at a 30 mM concentration. Treatment with this compound was carried out at times and concentrations indicated in Results and Figures. Control cells were exposed to an equivalent amount of vehicle. Cell counts were performed at 24 h intervals using the Trypan blue exclusion assay. All the experiments were repeated at least three times.

### siRNA transfection

Experiments of mRNA silencing were conducted transfecting a mix of three different siRNA oligonucleotides directed against TCF4 or CDX2 (Sigma-Aldrich) at a concentration of 100 nM with the RNAiMAX reagent (Invitrogen, Carlsbad, CA, USA). The transfection efficiency of CaCo2 cells was potentiated using the reverse transfection procedure.

### RNA extraction and QRT-PCR

Cells undergoing RNA extraction were trypsinized, washed once in PBS (EuroClone), re-suspended in lysis buffer and extracted with Qiagen total RNA purification kits (Qiagen, Valencia, CA, USA), following the manufacturer instructions. Total RNA was quantified using a NanoDrop 2000 spectrophotometer (Thermo Fisher Scientific, Waltham, MA, USA) and 100 ng were used to synthesize cDNA with the High Capacity cDNA Retro-transcription Kit (Invitrogen). Quantitative Real-Time PCR (QRT-PCR) was then conducted using an ABI PRISM 7900 detection system (Applied Biosystems). All primers and probes used for mRNA amplification were designed by Applied Biosystems. Each cDNA sample was run in triplicate using the Taqman Universal PCR Master Mix (Invitrogen) and glyceraldehyde-3-phosphate dehydrogenase was used as an endogenous control. Quantification of RT-PCR signals was performed using the Ct relative quantification method. This procedure calculates the relative changes in gene expression of the target gene normalized with an endogenous control and compared with a calibrator sample.

### Protein extract preparation and western blot analysis

Preparation of nuclear and cytoplasmic protein extracts and western blot analysis were carried out as previously described.^19^ In brief, electrophoresis was performed on sodium dodecyl sulfate polyacrylamide gel electrophoresis followed by electro-blotting to nitrocellulose sheets. Blotted membranes were pre-blocked with a solution containing 5% nonfat milk (Regilait, Saint Martin-Belle-Roche, France) in 0.05% TBST and incubated with a primary antibody specific for each analyzed protein, and with a common secondary antibody conjugated to horseradish peroxidase.

The following primary antibodies and the respective dilutions were used for western blot analysis: mouse anti-MUCDHL monoclonal antibody (MoAb) (A-11, Santa Cruz Biotechnology, Santa Cruz, CA, USA) 1:500 in TBST 3% milk; rabbit anti-CDX2 MoAb (ab76541 Abcam, Cambridge, UK) 1:1000 in TBST 1% milk; mouse anti-E-Cadherin MoAb (610181 BD Transduction Laboratories, San Jose, CA, USA) 1:1000 in TBST 5% milk; rabbit anti-TCF4 MoAb (C48H11 Cell Signaling Technology, Danvers, MA, USA) 1:1000 in TBST 5% BSA.

Expression of actin was also evaluated with a mouse pan-actin MoAb (Millipore Corporation, Billerica, MA, USA) to normalize the protein content of the various analyzed samples.

As secondary antibodies, a goat anti-mouse IgG (Santa Cruz Biotechnology) or a goat anti-rabbit IgG (Cell Signaling Technology), both conjugated with horseradish peroxidase, were used respectively at 1:10 000 and 1:15 000 dilutions. Detection of western blot signals was carried out using the Westar EtaC enhanced chemi-luminescent substrate (Cyanagen S.r.l., Bologna, Italy). Densitometric analysis was accomplished using the ImageJ Software.

### Culture of colonic organoids

Samples of normal colon mucosa were obtained from the Bio-Bank of Modena (BBM) (C.O.M. Via del Pozzo 71 41124, Modena, Italy). After surgical resection, the cancerous mucosa was used for histopathological analysis and for the BBM sampling, whereas a small fragment of healthy tissue was used for experiments and the rest was discharged.

Culture of human colonic organoids was carried out as described by Peter Jung.^9^ In brief, colonic crypts were separated from the underlying stroma through chelating agents, washed thoroughly and embedded in matrigel. After matrigel polymerization, the crypts were overlaid with complete growth medium which was composed of HAM's F12 supplemented with: antibiotics, 5% FBS (Sigma-Aldrich), Glutamax 1 × (Life Technologies), 10 mM HEPES, N-2 Supplement (Life Technologies) 1 ×, B-27 supplement without retinoic acid (Life Technologies) 1 × , 10 mM Nicotinamide (Sigma-Aldrich), 1 mM *N*-Acetyl-l-cysteine (Sigma-Aldrich), 1 *μ*g/ml RSPO1 (R&D System, McKinley Place NE, Minneapolis), 100 ng/ml human EGF (R&D System), 100 ng/ml human Noggin (R&D System), 1 *μ*g/ml Gastrin I (Sigma), 100 ng/ml WNT3a (R&D System), 500 nM LY2157299 (Axon MedChem, Groningen, The Netherlands), 10 *μ*M SB202190 (Sigma), and 0.01 *μ*M PGE2 (Sigma). For the first 48–72 h of culture, the crypts were left in complete growth medium, with the addition of the Rock inhibitor Y-27623 (10 *μ*M Sigma-Aldrich), in order to allow the formation of organoids.

In the following days, they were subjected to various treatments. Incubation with LiCl (Sigma) was conducted at 5 and 10 mM concentrations of the compound. Reduced levels of WNT3a were obtained lowering its concentration to 20 or 10 ng/ml, corresponding to 1/5 and 1/10 of the standard amount. The differentiation condition was achieved in complete absence of WNT3A, PGE2 and SB202190.

After 72 h of treatment, organoids were collected from matrigel with Cell Dissociation Solution (BD Transduction), washed with cold PBS and RNA was extracted as previously described.

Morphological analysis of organoids was conducted using the ImageJ software on pictures obtained by light-inverted microscope examination. Circularity was assessed outlining their outer edge with the freehand selection tool and expressed as Units (U) (1 U representing the value generated by a perfect circle), whereas the thickness of their epithelial lining was measured using the straight line selection tool, and expressed in micrometers (*μ*m).

### Statistical analysis

All experiments were repeated at least three times, unless otherwise stated, and the results presented in terms of mean±S.E.M. values. Pairwise comparisons were carried out using the Student's *t*-test procedure. Results of statistical analysis were considered significant at *P-*values <0.05 (*<0.05, **<0.001, ***<0.0001).

## Abbreviations

MUCDHL: *μ*-protocadherin
CRC: colorectal cancer
5-ASA: mesalazine
TCF4: transcription factor 7-like 2
CDX2: caudal type homeobox transcription factor 2
QRT-PCR: quantitative real-time PCR
GAPDH: glyceraldehyde-3-phosphate dehydrogenase
BBM: Bio-Bank of Modena
RSPO1: R-spondin 1
EGF: epidermal growth factor
WNT3a: wingless-Type MMTV Integration Site Family, Member 3A
PGE2: prostaglandin E2
APC: adenomatous polyposis coli
Snail: snail family zinc finger 1
ITF2: transcription factor 4
Twist: twist family bHLH transcription factor 1
Slug: snail family zinc finger 2
GSK3*β*: glycogen synthase kinase 3 beta
Zeb1: zinc finger E-box binding homeobox 1
