# Supplementary material for: Expression of μ-protocadherin is negatively regulated by the activation of the β-catenin signaling pathway in normal and cancer colorectal enterocytes
Source: Cell Death Dis. 2016 Jun 16;7(6):e2263–. doi: 10.1038/cddis.2016.163 (PMC5143391; doi:10.1038/cddis.2016.163)
Supplement: Supplementary Figure 1 Legend [file cddis2016163x2.doc]

**Supplementary figure 1.** Hypothetical mechanisms explaining the effects of 5-ASA on μ-protocadherin and β-catenin. In both hypotheses, 5-ASA is responsible for μ-protocadherin induction and β-catenin inhibition. In Hypothesis 1, the former is the cause of the latter, whereas in Hypothesis 2 it is the effect. Moreover, the ability of μ-protocadherin to sequester and inhibit β-catenin, in Hypothesis 1, would represent the main mechanism of β-catenin inhibition whereas, in Hypothesis 2 (supported by our data), it would represent a positive feed-back potentiating the final effect.
