# Supplementary material for: Expression of μ-protocadherin is negatively regulated by the activation of the β-catenin signaling pathway in normal and cancer colorectal enterocytes
Source: Cell Death Dis. 2016 Jun 16;7(6):e2263–. doi: 10.1038/cddis.2016.163 (PMC5143391; doi:10.1038/cddis.2016.163)
Supplement: Supplementary Table 1 [file cddis2016163x3.doc]

# Supplementary Table 1. Analysis of cell proliferation performed in CaCo2 cells upon treatment with different concentrations of FH535. Results are reported as number of cells expansions together with their and SEM (Standard Error of the Mean) and p values.

| **Cells expansions** | | | | | |
| --- | --- | --- | --- | --- | --- |
| FH535 | 0 h | 24 h | 48 h | 72 h | 96 h |
| 0 μM | 1 | 1.7 | 1.9 | 2.4 | 3.9 |
| 7.5 μM | 1 | 1.1 | 2.1 | 2.0 | 2.4 |
| 15 μM | 1 | 1.0 | 1.7 | 1.7 | 1.4 |
| 30 μM | 1 | 0.8 | 1.0 | 1.2 | 0.8 |
| 60 μM | 1 | 0.6 | 0.9 | 0.7 | 0.7 |
| **SEM** | | | | | |
| FH535 | 0 h | 24 h | 48 h | 72 h | 96 h |
| 0 μM | 0 | 0.5 | 0.2 | 0.2 | 0.6 |
| 7.5 μM | 0 | 0.2 | 0.5 | 0.2 | 0.5 |
| 15 μM | 0 | 0.1 | 0.4 | 0.4 | 0.1 |
| 30 μM | 0 | 0.1 | 0.1 | 0.2 | 0.0 |
| 60 μM | 0 | 0.1 | 0.1 | 0.2 | 0.2 |
| **p values** | | | | | |
| FH535 | 0 h | 24 h | 48 h | 72 h | 96 h |
| 0 μM | - | - | - | - | - |
| 7.5 μM | 0 | 0.3397 | 0.6771 | 0.1855 | 0.1294 |
| 15 μM | 0 | 0.2491 | 0.7460 | 0.1825 | 0.0510 |
| 30 μM | 0 | 0.2007 | 0.0327 | 0.0081 | 0.0383 |
| 60 μM | 0 | 0.1280 | 0.0264 | 0.0016 | 0.0248 |
