# Supplementary material for: Expression of μ-protocadherin is negatively regulated by the activation of the β-catenin signaling pathway in normal and cancer colorectal enterocytes
Source: Cell Death Dis. 2016 Jun 16;7(6):e2263–. doi: 10.1038/cddis.2016.163 (PMC5143391; doi:10.1038/cddis.2016.163)
Supplement: Supplementary Table 3 [file cddis2016163x5.doc]

# Supplementary Table 3. Analysis of mRNA expression performed by qRT-PCR in CaCo2 cells exposed to different concentrations of FH535. Results are reported as fold change together with their SEM and p values.

| **Fold change** | | | | |
| --- | --- | --- | --- | --- |
| FH535 | MUCDHL | CDH1 | p21 ^waf1^ | CDX2 |
| 0 μM | 1 | 1 | 1 | 1 |
| 30 μM | 1.9 | 1.3 | 5.0 | 1.6 |
| 60 μM | 3.4 | 2.2 | 8.6 | 1.9 |
| **SEM** | | | | |
| FH535 | MUCDHL | CDH1 | p21 ^waf1^ | CDX2 |
| 0 μM | 1 | 1 | 1 | 1 |
| 30 μM | 0.2 | 0.1 | 1.8 | 0.1 |
| 60 μM | 0.2 | 0.1 | 1.0 | 0.0 |
| **p values** | | | | |
| FH535 | MUCDHL | CDH1 | p21 ^waf1^ | CDX2 |
| 0 μM | - | - | - | - |
| 30 μM | 0.0682 | 0.0450 | 0.2696 | 0.0160 |
| 60 μM | 0.0081 | 0.0666 | 0.0176 | 0.0231 |
