# Supplementary material for: Expression of μ-protocadherin is negatively regulated by the activation of the β-catenin signaling pathway in normal and cancer colorectal enterocytes
Source: Cell Death Dis. 2016 Jun 16;7(6):e2263–. doi: 10.1038/cddis.2016.163 (PMC5143391; doi:10.1038/cddis.2016.163)
Supplement: Supplementary Table 4 [file cddis2016163x6.doc]

# Supplementary Table 4. Analysis of mRNA expression performed by qRT-PCR in HCT116 cells exposed to different concentrations of FH535. Results are reported as fold change together with their SEM and p values.

| **Fold change** | | | | |
| --- | --- | --- | --- | --- |
| FH535 | MUCDHL | CDH1 | p21 ^waf1^ | CDX2 |
| 0 μM | 1.0 | 1.0 | 1.0 | 1.0 |
| 15 μM | 1.4 | 1.8 | 2.0 | 0.9 |
| 30 μM | 3.2 | 5.0 | 11.5 | 10.3 |
| **SEM** | | | | |
| FH535 | MUCDHL | CDH1 | p21 ^waf1^ | CDX2 |
| 0 μM | 0.0 | 0.0 | 0.0 | 0.0 |
| 15 μM | 0.2 | 0.4 | 0.5 | 0.2 |
| 30 μM | 0.6 | 1.5 | 4.0 | 8.0 |
| **p values** | | | | |
| FH535 | MUCDHL | CDH1 | p21 ^waf1^ | CDX2 |
| 0 μM | - | - | - | - |
| 15 μM | 0.2578 | 0.1148 | 0.1117 | 0.5845 |
| 30 μM | 0.1695 | 0.1218 | 0.1188 | 0.4502 |
