# Supplementary material for: Expression of μ-protocadherin is negatively regulated by the activation of the β-catenin signaling pathway in normal and cancer colorectal enterocytes
Source: Cell Death Dis. 2016 Jun 16;7(6):e2263–. doi: 10.1038/cddis.2016.163 (PMC5143391; doi:10.1038/cddis.2016.163)
Supplement: Supplementary Table 5 [file cddis2016163x7.doc]

# Supplementary Table 5. Analysis of mRNA expression performed by qRT-PCR in CaCo2 cells transfected with anti - TCF4 specific (TCF4) or scrambled (Cont.) oligonucleotide siRNAs. Results are reported as fold change together with their SEM and p values.

| **Fold change** | | | | |
| --- | --- | --- | --- | --- |
|  | TCF4 | CDX2 | MUCDHL | p21 ^waf1^ |
| Cont. siRNA | 1 | 1 | 1 | 1 |
| TCF4 siRNA | 0.7 | 1.9 | 1.9 | 1.6 |
| **SEM** | | | | |
|  | TCF4 | CDX2 | MUCDHL | p21 ^waf1^ |
| Cont. siRNA | 0 | 0 | 0 | 0 |
| TCF4 siRNA | 0.1 | 0.2 | 0.2 | 0.1 |
| **p values** | | | | |
|  | TCF4 | CDX2 | MUCDHL | p21 ^waf1^ |
| Cont. siRNA | - | - | - | - |
| TCF4 siRNA | 0.0907 | 0.0175 | 0.0298 | 0.0081 |
