# Supplementary material for: Expression of μ-protocadherin is negatively regulated by the activation of the β-catenin signaling pathway in normal and cancer colorectal enterocytes
Source: Cell Death Dis. 2016 Jun 16;7(6):e2263–. doi: 10.1038/cddis.2016.163 (PMC5143391; doi:10.1038/cddis.2016.163)
Supplement: Supplementary Table 7 [file cddis2016163x9.doc]

# Supplementary Table 7. Analysis of mRNA expression performed by qRT-PCR in normal colonic organoids undergoing Wnt deprivation. Colonic organoids were cultured in presence of reduced concentrations of WNT3a (1/5 and 1/10 Wnt) or in complete absence of β-catenin pathway agonists (Differentiation conditions). Results are reported as fold change together with their SEM and p values.

| **Fold change** | | | | | | | |
| --- | --- | --- | --- | --- | --- | --- | --- |
|  | MUCDHL | KRT20 | CDH1 | CDX2 | p21 ^waf1^ | MET | CD44 |
| Cont. | 1 | 1 | 1 | 1 | 1 | 1 | 1 |
| 1/5 Wnt | 1.9 | 1.7 | 1.3 | 1.6 | 1.2 | 0.7 | 0.4 |
| 1/10 Wnt | 2.4 | 1.8 | 1.0 | 1.8 | 1.4 | 0.7 | 0.3 |
| Differentiation conditions | 3.4 | 3.2 | 1.6 | 2.5 | 3.8 | 0.8 | 0.3 |
| **SEM** | | | | | | | |
|  | MUCDHL | KRT20 | CDH1 | CDX2 | p21 ^waf1^ | MET | CD44 |
| Cont. | 0 | 0 | 0 | 0 | 0 | 0 | 0 |
| 1/5 Wnt | 0.2 | 0.3 | 0.1 | 0.1 | 0.1 | 0.1 | 0.0 |
| 1/10 Wnt | 0.3 | 0.1 | 0.3 | 0.3 | 0.3 | 0.3 | 0.0 |
| Differentiation conditions | 0.6 | 0.5 | 0.1 | 0.5 | 0.9 | 0.1 | 0.1 |
| **p values** | | | | | | | |
|  | MUCDHL | KRT20 | CDH1 | CDX2 | p21 ^waf1^ | MET | CD44 |
| Cont. | - | - | - | - | - | - | - |
| 1/5 Wnt | 0.0242 | 0.1056 | 0.0592 | 0.0337 | 0.0503 | 0.0301 | 0.0001 |
| 1/10 Wnt | 0.0427 | 0.0150 | 0.9787 | 0.1353 | 0.3837 | 0.4050 | 0.0013 |
| Differentiation conditions | 0.0204 | 0.0107 | 0.0025 | 0.0194 | 0.0518 | 0.0538 | 0.0003 |
