# Supplementary material for: Expression of μ-protocadherin is negatively regulated by the activation of the β-catenin signaling pathway in normal and cancer colorectal enterocytes
Source: Cell Death Dis. 2016 Jun 16;7(6):e2263–. doi: 10.1038/cddis.2016.163 (PMC5143391; doi:10.1038/cddis.2016.163)
Supplement: Supplementary Table 8 [file cddis2016163x10.doc]

# Supplementary Table 8. Analysis of mRNA expression performed by qRT-PCR in normal colonic organoids undergoing treatment with different LiCl concentrations. Results are reported as fold change together with their SEM and p values.

| **Fold change** | | | | | | | |
| --- | --- | --- | --- | --- | --- | --- | --- |
|  | MUCDHL | KRT20 | CDH1 | CDX2 | p21 ^waf1^ | MET | CD44 |
| Cont. | 1 | 1 | 1 | 1 | 1 | 1 | 1 |
| LiCl 5 mM | 0.6 | 0.7 | 0.6 | 0.5 | 0.6 | 1.1 | 2.2 |
| LiCl 10 mM | 0.5 | 0.4 | 0.5 | 0.4 | 0.6 | 0.9 | 2.6 |
| **SEM** | | | | | | | |
|  | MUCDHL | KRT20 | CDH1 | CDX2 | p21 ^waf1^ | MET | CD44 |
| Cont. | 0 | 0 | 0 | 0 | 0 | 0 | 0 |
| LiCl 5 mM | 0.1 | 0.1 | 0.1 | 0.1 | 0.1 | 0.2 | 0.4 |
| LiCl 10 mM | 0.0 | 0.1 | 0.1 | 0.1 | 0.1 | 0.0 | 0.2 |
| **p values** | | | | | | | |
|  | MUCDHL | KRT20 | CDH1 | CDX2 | p21 ^waf1^ | MET | CD44 |
| Cont. | - | - | - | - | - | - | - |
| LiCl 5 mM | 0.0142 | 0.0410 | 0.0127 | 0.0374 | 0.0117 | 0.7787 | 0.0633 |
| LiCl 10 mM | 0.0082 | 0.0102 | 0.0446 | 0.0218 | 0.0280 | 0.1122 | 0.0206 |
