# Supplementary material for: Expression of μ-protocadherin is negatively regulated by the activation of the β-catenin signaling pathway in normal and cancer colorectal enterocytes
Source: Cell Death Dis. 2016 Jun 16;7(6):e2263–. doi: 10.1038/cddis.2016.163 (PMC5143391; doi:10.1038/cddis.2016.163)
Supplement: Supplementary Table 9 [file cddis2016163x11.doc]

# Supplementary Table 9. Analysis of mRNA expression performed by qRT-PCR in CaCo2 cells transfected with anti – CDX2 specific (CDX2) or scrambled (Cont.) oligonucleotide siRNAs and subsequently treated with 5-ASA 20mM (5-ASA) or untreated (N.T.). Results are reported as fold change together with their SEM and p values.

| **Fold change** | | |
| --- | --- | --- |
|  | MUCDHL | CDX2 |
| Cont. siRNA N.T. | 1 | 1 |
| Cont. siRNA + 5-ASA | 2.9 | 3.4 |
| CDX2 siRNA N.T. | 1.4 | 0.4 |
| CDX2 siRNA+ 5-ASA | 1.8 | 1.4 |
| **SEM** | | |
|  | MUCDHL | CDX2 |
| Cont. siRNA N.T. | 0 | 0 |
| Cont. siRNA + 5-ASA | 0.6 | 0.1 |
| CDX2 siRNA N.T. | 0.2 | 0.0 |
| CDX2 siRNA+ 5-ASA | 0.5 | 0.2 |
| **p values** | | |
|  | MUCDHL | CDX2 |
| Cont. siRNA N.T. | - | - |
| Cont. siRNA + 5-ASA | 0.1938 | 0.0132 |
| CDX2 siRNA N.T. | 0.2563 | 0.0269 |
| CDX2 siRNA+ 5-ASA | 0.3621 | 0.3262 |
