# Supplementary material for: Expression of μ-protocadherin is negatively regulated by the activation of the β-catenin signaling pathway in normal and cancer colorectal enterocytes
Source: Cell Death Dis. 2016 Jun 16;7(6):e2263–. doi: 10.1038/cddis.2016.163 (PMC5143391; doi:10.1038/cddis.2016.163)
Supplement: Supplementary Table 10 [file cddis2016163x12.doc]

# Supplementary Table 10. Analysis of the circularity of colonic organoids cultured under differentiation conditions. Circularity of colonic organoids cultured under standard conditions (Cont.) or differentiation conditions (Diff. Conditions) was measured as described in Materials and Methods and expressed as Units. The table shows the values obtained from two independent experiments.

|  | Cont. (U) | Diff.  Conditions (U) |
| --- | --- | --- |
| 1 | 0.919 | 0.885 |
| 2 | 0.912 | 0.735 |
| 3 | 0.909 | 0.679 |
| 4 | 0.852 | 0.860 |
| 5 | 0.947 | 0.874 |
| 6 | 0.867 | 0.709 |
| 7 | 0.857 | 0.840 |
| 8 | 0.908 | 0.763 |
| 9 | 0.880 | 0.819 |
| 10 | 0.891 | 0.754 |
| 11 | 0.883 | 0.834 |
| 12 | 0.908 | 0.627 |
| 13 | 0.860 | 0.837 |
| 14 | 0.831 | 0.722 |
| 15 | 0.766 | 0.828 |
| 16 | 0.889 | 0.800 |
| 17 | 0.821 | 0.772 |
| 18 | 0.897 | 0.930 |
| 19 | 0.889 | 0.874 |
| 20 | 0.886 | 0.677 |
| 21 | 0.833 | 0.803 |
| 22 | 0.890 | 0.834 |
| 23 | 0.926 | 0.653 |
| 24 | 0.837 | 0.861 |
| 25 | 0.911 | 0.614 |
| 26 | 0.838 | 0.874 |
| 27 | 0.869 | 0.765 |
| 28 | 0.742 | 0.812 |
| 29 | 0.869 | 0.739 |
| 30 | 0.936 | 0.835 |
| 31 | 0.901 | 0.873 |
| 32 | 0.863 | 0.766 |
| 33 | 0.819 | 0.884 |
| 34 | 0.877 | 0.808 |
| 35 | 0.812 | 0.877 |
| 36 | 0.947 | 0.581 |
| 37 | 0.955 | 0.896 |
| 38 | 0.851 | 0.937 |
| 39 | 0.931 | 0.706 |
| 40 | 0.912 | 0.696 |
| 41 | 0.845 | 0.846 |
| 42 | 0.720 | 0.772 |
| 43 | 0.934 | 0.726 |
| 44 | 0.928 | 0.871 |
| 45 | 0.907 | 0.805 |
| 46 | 0.882 | 0.683 |
| 47 | 0.921 | 0.816 |
| 48 | 0.774 |  |
| 49 | 0.860 |  |
| 50 | 0.883 |  |
| 51 | 0.893 |  |
| 52 | 0.801 |  |
| 53 | 0.951 |  |
| 54 | 0.925 |  |
| 55 | 0.899 |  |
