# Supplementary material for: Expression of μ-protocadherin is negatively regulated by the activation of the β-catenin signaling pathway in normal and cancer colorectal enterocytes
Source: Cell Death Dis. 2016 Jun 16;7(6):e2263–. doi: 10.1038/cddis.2016.163 (PMC5143391; doi:10.1038/cddis.2016.163)
Supplement: Supplementary Table 11 [file cddis2016163x13.doc]

# Supplementary Table 11. Analysis of the thickness of epithelial lining of colonic organoids treated with LiCl. The thickness of the epithelial lining of colonic organoids cultured under standard conditions (Cont.) or in presence of 10 mM LiCL (LiCL) was measured as described in Materials and Methods and expressed as μM. The table shows the values obtained from two independent experiments.

|  | Cont. (μm) | LiCl (μm) |
| --- | --- | --- |
| 1 | 14.462 | 59.101 |
| 2 | 16.377 | 25.637 |
| 3 | 16.967 | 48.444 |
| 4 | 14.462 | 46.439 |
| 5 | 17.255 | 48.207 |
| 6 | 23.529 | 53.128 |
| 7 | 36.078 | 73.835 |
| 8 | 25.098 | 42.927 |
| 9 | 25.098 | 34.526 |
| 10 | 17.255 | 39.794 |
| 11 | 23.529 | 42.000 |
| 12 | 28.279 | 71.050 |
| 13 | 19.655 | 35.770 |
| 14 | 25.147 | 46.451 |
| 15 | 21.961 | 62.763 |
| 16 | 10.226 | 54.830 |
| 17 | 12.549 | 48.207 |
| 18 | 37.940 | 68.869 |
| 19 | 38.964 | 65.341 |
| 20 | 26.713 | 34.191 |
| 21 | 25.147 | 30.327 |
| 22 | 25.147 | 38.455 |
| 23 | 14.118 | 44.395 |
| 24 | 14.462 | 75.702 |
| 25 | 19.655 | 47.163 |
| 26 | 17.778 | 57.250 |
| 27 | 19.648 | 27.079 |
| 28 | 14.789 | 34.259 |
| 29 | 13.635 | 53.609 |
| 30 | 19.452 | 126.411 |
| 31 | 31.390 | 35.556 |
| 32 | 10.510 | 59.818 |
| 33 | 10.510 | 61.085 |
| 34 | 48.100 | 44.760 |
| 35 | 21.045 | 55.375 |
| 36 | 25.098 | 48.647 |
| 37 | 20.452 | 82.023 |
| 38 | 28.235 | 43.971 |
| 39 | 21.961 | 41.673 |
| 40 | 23.319 | 49.890 |
| 41 | 40.905 | 31.994 |
| 42 | 19.857 | 22.294 |
| 43 | 28.235 | 42.237 |
| 44 | 25.727 | 65.545 |
| 45 | 31.136 | 36.113 |
| 46 | 30.457 |  |
| 47 | 65.901 |  |
| 48 | 24.857 |  |
| 49 | 17.809 |  |
